# Limits on Monolingualism? A Comparison of Monolingual and Bilingual Infants’ Abilities to Integrate Lexical Tone in Novel Word Learning

**DOI:** 10.3389/fpsyg.2016.00667

**Published:** 2016-05-10

**Authors:** Leher Singh, Felicia L. S. Poh, Charlene S. L. Fu

**Affiliations:** Department of Psychology, National University of Singapore, SingaporeSingapore

**Keywords:** lexical tone, phoneme discrimination, infant speech perception, Mandarin Chinese, word learning

## Abstract

To construct their first lexicon, infants must determine the relationship between native phonological variation and the meanings of words. This process is arguably more complex for bilingual learners who are often confronted with phonological conflict: phonological variation that is lexically relevant in one language may be lexically irrelevant in the other. In a series of four experiments, the present study investigated English–Mandarin bilingual infants’ abilities to negotiate phonological conflict introduced by learning both a tone and a non-tone language. In a novel word learning task, bilingual children were tested on their sensitivity to tone variation in English and Mandarin contexts. Their abilities to interpret tone variation in a language-dependent manner were compared to those of monolingual Mandarin learning infants. Results demonstrated that at 12–13 months, bilingual infants demonstrated the ability to bind tone to word meanings in Mandarin, but to disregard tone variation when learning new words in English. In contrast, monolingual learners of Mandarin did not show evidence of integrating tones into word meanings in Mandarin at the same age even though they were learning a tone language. However, a tone discrimination paradigm confirmed that monolingual Mandarin learning infants were able to tell these tones apart at 12–13 months under a different set of conditions. Later, at 17–18 months, monolingual Mandarin learners were able to bind tone variation to word meanings when learning new words. Our findings are discussed in terms of cognitive adaptations associated with bilingualism that may ease the negotiation of phonological conflict and facilitate precocious uptake of certain properties of each language.

## Introduction

Languages of the world make use of sound in different ways to create words. A classic example is the use of vocal pitch in human languages. When learning a tone language like Mandarin Chinese, listeners must register particular changes in vocal pitch that distinguish the meanings of words. However, pitch variation is also a ubiquitous feature of non-tone languages such as English and is used to distinguish questions/statements, emotional states, and placement of stress and focus. In contrast to Mandarin learners, English learners must disregard pitch variation when determining the lexical identity of a word. It is therefore incumbent upon the young language learner to determine how sound changes effect changes in word meaning in their native language to construct a vocabulary. By necessity, children learning two languages have to learn how words are defined in both of their native languages. This process is potentially complicated by the fact that the phonological rules of two languages can diverge as in the case of Mandarin and English where pitch varies lexically and non-lexically, respectively, causing a potential conflict. The purpose of the current study is to determine how bilingual infants resolve this conflict and negotiate cross-language phonological conflict when learning new words. Specifically, the present study focuses on English–Mandarin bilingual infants’ abilities to define words according to lexical tone when listening to Mandarin and to disregard the same source of variation in pitch when defining new words in English. Bilingual infants’ abilities to integrate pitch in a language-dependent fashion are interpreted in relation to those of monolingual tone language learners.

In prior research, children’s abilities to integrate native phonological variation when learning new words have been widely studied in monolingual children (Stager and Werker, 1997; Pater et al., 2004; Dietrich et al., 2007; Rost and McMurray, 2009, 2010; Yoshida et al., 2009), but to a much lesser extent in bilingual children (but see Fennell et al., 2007; Mattock et al., 2010; Byers-Heinlein et al., 2013; Fennell and Byers-Heinlein, 2014). A substantial proportion of this research has used the Switch task, which has been productively used to investigate infants’ abilities to map similar sounding words onto different meanings. In a common instantiation of this task, infants are familiarized with an on-screen display of two objects and their labels. Labels consist of novel words that are subtle phonemic variants – or minimal pairs (e.g., ‘bih’ and “dih”). During a habituation phase, infants are presented with repetitions of each pairing until their attention to the objects wanes to a pre-set criterion. Following the habituation phase, infants are presented with two test trials. In one test trial (Same trial), infants are presented with the pairing with which they were familiarized. In the other test trial (Switch trial), infants are presented with the visual object with which they were familiarized but it is labeled with the name for the other object (e.g., what was learned as a ‘bih’ is now labeled as a ‘dih’). Infants’ fixation times to each trial type are compared: a relative elevation in fixation to the Switch trial versus the Same trial is interpreted as evidence of infants’ sensitivity to the source of phonological variation incorporated into the task (i.e., to variation in place of articulation in the current example).

In a seminal study that pioneered the Switch task to investigate early word learning, Stager and Werker (1997) demonstrated that 14-month-old monolingual infants failed to incorporate phonological variation (i.e., the difference between ‘b-’ versus ‘d-’) when learning new words, although they could incorporate the same variation when recognizing familiar words (Fennell and Werker, 2003). Comparative studies with bilingual infants reveal a similar set of abilities provided that bilingual infants are provided with input that is consistent with the phonetic properties of their input (i.e., input that sounds native to them). In one such study by Mattock et al. (2010), the authors presented 17-month-old bilingual infants with tokens drawn from both of their languages. Mattock et al. (2010) demonstrated that under these conditions, bilingual infants linked similar sounding words to their meanings at 17 months. More recently, Fennell and Byers-Heinlein (2014) demonstrated that both 17-month-old monolingual and bilingual infants succeeded in learning similar sounding words when the speaker matched their language background (i.e., when the speaker was monolingual or bilingual, respectively), although bilingual infants were not able to learn similar sounding words when presented with monolingual input (see Fennell et al., 2007). In sum, this set of studies suggests that both 17- to 18-month-old monolingual and bilingual infants maintain keen perceptual sensitivities to subtle phonetic detail that are optimally engaged when they listen to language input reminiscent of their environment.

Previous research has focused on bilingual infants’ sensitivity to phonological variation that draws lexical distinctions in both of their native languages (although the sub-phonetic realization of these sounds varies across languages; e.g., Fennell et al., 2007; Mattock et al., 2010; Fennell and Byers-Heinlein, 2014). Nevertheless, in each of the aforementioned studies, the phonemes used to distinguish word meanings belonged to separate phonetic categories in *both* languages. However, bilinguals often have to negotiate phonological conflict where the same source of variation draws lexical distinctions only in one language and not in the other. In this situation, learners of two languages have to alternate between activating and de-activating sensitivity to this source of variation depending on the language in use. For example, learners of Mandarin–Chinese and English have to inhibit integration of pitch variation when defining new words in English but have to incorporate certain forms of pitch variation (i.e., tone contrasts) when learning new words in Mandarin. One prior study has investigated bilingual English–Mandarin infants’ abilities to integrate tone in English and Mandarin in a language-selective manner. In a word segmentation task investigating how effectively infants segment words from passages, Singh and Foong (2012) familiarized infants with isolated words and then tested infants’ recognition of the familiarized words in fluent speech. Each infant was tested in English and in Mandarin in succession. The critical manipulation was that in the test phase, the target word was either matched or mis-matched in tone (Mandarin session) or matched or mis-matched in pitch (English session). Infants were tested at 7.5-, 9-, and 11-months. While infants did not demonstrate language-selective integration of pitch at 7.5- and 9-months (either integrating pitch/tone variation or disregarding pitch/tone variation in both languages), at 11 months, infants selectively defined words by tone in Mandarin and not by pitch in English. However, this study did not involve forming word-object associations, as it was an auditory-only word segmentation task, rendering it unclear as to whether infants linked the familiarized words to meaning. Additionally, the pitch transformations qualitatively differed between English and Mandarin sessions: Mandarin pitch variants encompassed Mandarin lexical tone contrasts, while English pitch variants were digitized, uniform transformations across the entire syllable. However, most crucially, word segmentation is thought to measure an infants’ ability to track repetitions of the same word and prior to 12 months, and is thought to precede an infants’ determination of meaning (Jusczyk and Aslin, 1995).

Subsequent studies investigating integration of pitch and tone when forming word-object associations reveal more fragile abilities in young children when they are required to link words to meaning. Influences of tone variation in newly learned words have been investigated in English monolingual infants, non-tone language learning bilingual infants and English–Mandarin bilingual infants (Singh et al., 2014; Graf Estes and Hay, 2015; Hay et al., 2015). Collectively, these findings suggest that the language-specific functions of pitch are not consolidated as early as 11 months. Using a preferential looking paradigm, a study by Singh et al. (2014) involved teaching infants novel tone-marked words in a referential context. Infants were then tested on their recognition of tone-matched and tone-varying labels of familiarized words (as well as vowel matches/variants). The authors reported that non-tone learning infants (monolingual and bilingual) were similar to their Mandarin learning peers in that they were sensitive to tone as a source of lexical contrast, rejecting tone variants as labels for words at 18 months. It was not until 24 months that non-tone learning infants (monolingual and bilingual) demonstrated selective inhibition to tone in English when learning new words, whereas Mandarin learning infants continued to associate and integrate lexical tone into newly learned words at 24 months. Tone integration was reflected by participant’s construal of tone changes as mispronunciations of newly learned words. In an investigation of tone sensitivity in English monolingual infants using the Switch paradigm, Hay et al. (2015) investigated English learning infants’ sensitivity to rising and falling tones when learning new words at 14, 17, and 19 months. Infants exhibited developmental change in tone sensitivity between 14 and 17–19 months: while 14-month-old infants were sensitive to tone variation, at 17 and 19 months, infants were no longer sensitive to the same source of tone variation in the Switch paradigm. Posing this question with bilingual infants learning two non-tone languages, Graf Estes and Hay (2015) reported a protracted period of tone sensitivity in bilingual learners, demonstrating that these infants were sensitive to lexical tones at 14 and 19 months, but not at 22 months. In the aggregate, it appears that when infants are confronted with the added burden of forming word-object associations, their sensitivity to phonological variation appears much more fragile than when they are simply tracking repetitions of words across time as in Singh and Foong’s study. However, in Singh et al. (2014), although tone learners were English–Mandarin bilinguals, the language context of newly learned words was not manipulated within bilingual participants. As such, it was not possible to examine whether bilingual participants could actually shift their interpretation of tone as befitted the language context. The ability on the part of bilingual learners to re-interpret the same phonetic information in a language-selective manner – termed perceptual switching – has been well researched in adult bilinguals (Flege and Eefting, 1987; Hazan and Boulakia, 1993; García-Sierra et al., 2009, 2012; Gonzales and Lotto, 2013) and to a limited extent, in children (Singh and Quam, 2016), but not yet in infants. However, this process of rapid alternation is a fundamental component of bilingual proficiency. The current study focuses on monolingual and bilingual infants’ abilities to alternate between the phonological systems of each of their languages when these systems conflict.

**FIGURE 1:**
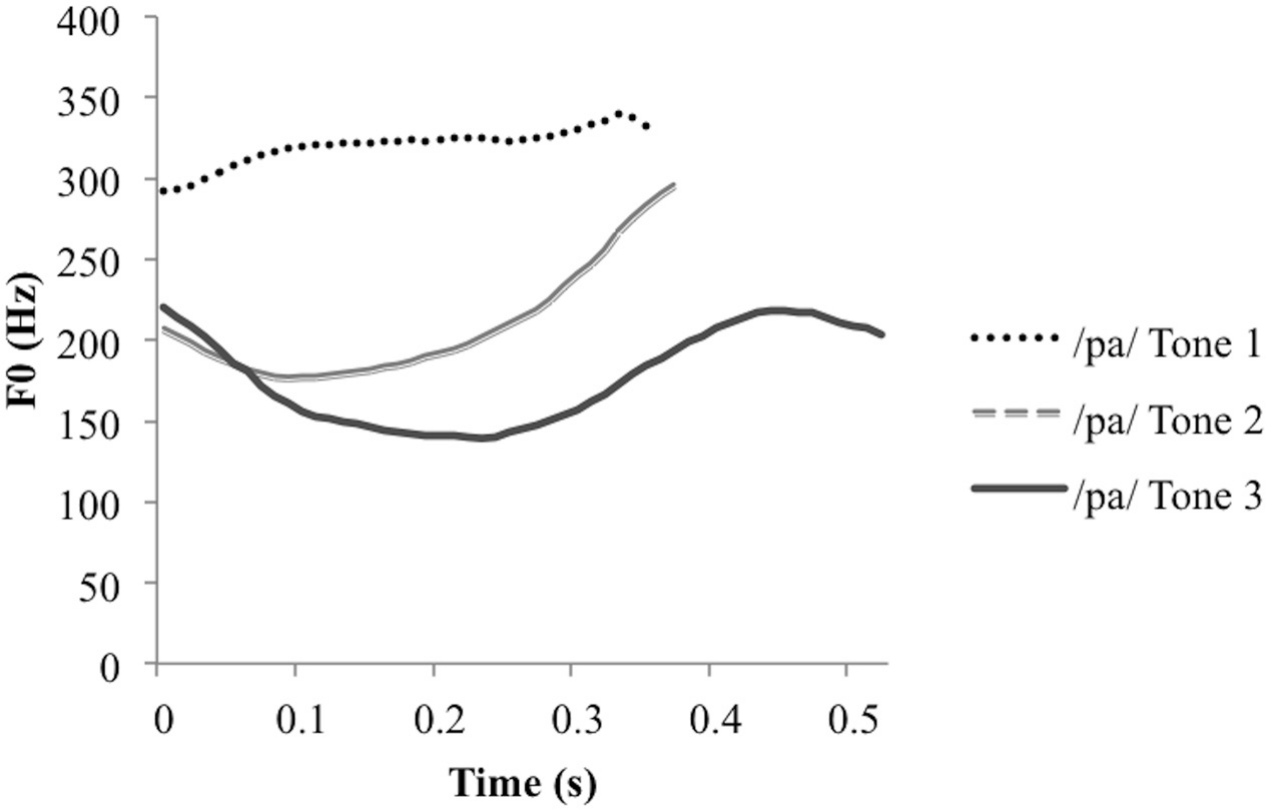
**Pitch contours for the three target syllables used in the habituation and test phases**.

The primary goal of this study is to compare monolingual and bilingual phonological representations of lexical tone by assessing infants’ responsiveness to tone mispronunciations in their native language(s). In light of the multi-functionality of pitch in English–Mandarin bilingual infants’ environments, infants were provided with naming phrases ending with target words to cue a particular language (i.e., English or Mandarin). Prior research has demonstrated that bilingual infants make productive use of naming phrases to identify the relevant phonological rules (see Fennell and Byers-Heinlein, 2011). A secondary goal of the present study was to determine whether sensitivity to a change in lexical tone depended not only on the language in use, but furthermore, on the acoustic salience of the tone change. Mandarin Chinese has four lexical tones [high (Tone 1), rising (Tone 2), dipping (Tone 3), falling (Tone 4)], three of which (Tones 1, 2, and 3) were used in our study (please see **Figure 1** for an illustration of Tones 1, 2, and 3). Some tones are highly distinctive from one another (such as Tones 1 and 3) such that Mandarin speakers readily discriminate them (Chen, 2013). Other tones are highly similar, notably Tones 2 and 3, such that these tones are often poorly discriminated (Zue, 1976; Shen and Lin, 1991). Prior studies investigating infants’ sensitivity to lexical tones have revealed that sensitivity to lexical tone pairs progresses asynchronously for different tone pairs (see Mattock and Burnham, 2006; Tsao, 2008; Yeung et al., 2013; Liu and Kager, 2014). An important determinant of lexical tone perception appears to be the salience of the tone contrast (see Liu and Kager, 2014 and Tsao, 2008 for investigations of sensitivity to high and low salience tone contrasts), a pattern also evidenced in production (e.g., Wong et al., 2005). Prior studies have demonstrated that emergent sensitivity to lexical tone contrasts do not necessarily generalize across the entire tone inventory (see Singh and Fu, 2016, for a review of this evidence in perception and production). Conclusions drawn about tone sensitivity are therefore necessarily qualified by the relative similarity of a given tone pair. Tone similarity is commonly defined by properties of the pitch contour (Gandour, 1983), primarily by pitch direction and secondarily by pitch height (Chandrasekaran et al., 2010). In light of discrepant effects of similar and distinct tone pairs on tone sensitivity, in the current study, infants’ sensitivity to lexical tone as a source of contrast was compared across similar and distinct tone pairs.

A series of four experiments are reported. In Experiment 1, 12–13-month-old bilingual English–Mandarin infants were tested on a similar task, but were tested in both Mandarin and English in direct succession. In Experiment 2, 12–13-month-old monolingual Mandarin learning infants were tested on their sensitivity to lexical tone contrasts when learning novel words in Mandarin. Experiments 3 and 4 were designed to further investigate the apparent insensitivity to lexical tone observed in Mandarin learning monolingual infants at 12–13 months. Experiment 3 investigated whether Mandarin learning monolingual infants could discriminate the tones used in Experiment 2, even though they did not appear sensitive to variation in these tones when learning novel words. Experiment 4 investigated whether Mandarin learning monolingual infants could integrate lexical tone contrasts at a later age, testing 18-month-old infants on the same word learning task administered to 12- to 13-month-old Mandarin infants in Experiment 2.

## Experiment 1

In Experiment 1, we investigated whether bilingual infants, learning English and Mandarin, integrated tone in a language-selective manner within each of their native languages. The purpose of this experiment was to determine whether habitual exposure to two native languages that conflicted in their use of tone would facilitate a language-selective interpretation of tone. We hypothesized that the contrastive use of tone in each of the participants’ native languages would contribute to a more mature understanding of the linguistic functions served by tone in each language.

Infants were familiarized with a word object pairing via the Switch paradigm. The label used to introduce the object was spoken in Tone 3. After successfully habituating to the pairing, infants were tested on their sensitivity to a similar (Tone 2 versus Tone 3) mispronunciation and to a distinct (Tone 1 versus Tone 3) mispronunciation. Infants were tested in each of their native languages: English and Chinese. Responses to each type of tone mispronunciation were compared across languages.

### Methods

#### Participants

Our sample comprised eighteen 12- to 13-month-old Mandarin–English bilingual infants (age range: 12 months 10 days to 13 months 21 days, average age = 13 months 1 day). All infants were born healthy and full term. Another seven infants were tested but excluded from the final sample due to fussiness during test (*n* = 6) or on account of data that deviated from the group mean by more than 3 standard deviations (*n* = 1). All infants received between at least 35% exposure to English or Mandarin with no third language exposure (range of English exposure: 38 to 63%, mean = 51%; range of Mandarin exposure: 37 to 62%; mean = 48%). Language exposure was determined by the Language Exposure Questionnaire developed by Bosch and Sebastián-Gallés (1997). Language exposure was derived from parental estimates of the relative proportion that each caregiver used when communicating directly to the child, and the amount of time each caregiver spent with the child in a typical week.

The age of testing was motivated by prior research investigating sensitivity to suprasegmental lexical variation (see Curtin, 2009). When tasked with learning minimally contrastive words differing in lexical stress, Curtin (2009) demonstrated that infants were sensitive to contrasts in stress at 1213 months. This finding stands in contrast to the broad swath of studies defining similar sounding words by consonant variation demonstrating that infants are challenged by this task prior to 14 months (e.g., Stager and Werker, 1997; Werker et al., 2002; Pater et al., 2004). As concluded by Curtin (2009), it appears that suprasegmental lexical variation is integrated into word meaning earlier than segmental variation. As our study manipulated suprasegmental lexical variation (i.e., tones), we tested infants at 12–13 months. This study was carried out with the approval of the National University of Singapore Institutional Review Board. Participants’ parents or legal guardians gave written informed consent in accordance with the National University of Singapore Institutional Review Board requirements.

#### Stimuli

Auditory stimuli for the study consisted of seven Mandarin and seven English naming phrases adapted from Fennell and Waxman (2010) (see **Table 1**). The target word was the label “pa” produced in Tones 1, 2, and 3 by a female native speaker of Mandarin in the context of each naming phrase. All stimuli were produced in infant-directed speech. The mean duration of the Mandarin phrases was 1.28 s (*SD*: 0.4) and the mean duration of the English phrases was 1.14 s (*SD*: 0.3). English and Mandarin phrase durations did not differ significantly. The mean pitch range of the carrier phrases was 288.14 Hz (*SD*: 45.81; Mandarin) and 277.95 Hz (*SD*: 48.51; English), which again, did not differ significantly across languages. The mean duration of the target words was 0.47 s (*SD*: 0.07). The same tokens were spliced into English and Mandarin introductory phrases to mitigate possible effects of language-specific differences in tone productions. Each instantiation of the target syllable was separated by 800 ms.

**Table 1:** Naming phrases used in Experiments 1, 2, and 4.

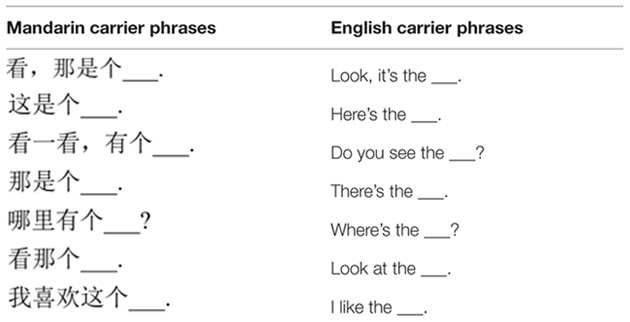

The target word was labeled by the syllable /pa/, which begins with an unaspirated voiceless onset consonant. This segment was chosen for the entire series of experiments because it assimilates to the native phonological inventories of English and Mandarin. In English, the unaspirated /p/ typically follows a word-initial /s/, such as in “spa,” but it does not appear in the word-initial position. However, unaspirated voiceless stops in word-initial position sound native to English speakers and are classified as voiced stops (in this case, ‘ba’; Pegg and Werker, 1997). They are judged to be as good an instance of ‘ba’ as the voiced stop ‘ba’ when produced in word-initial position (Lisker and Abramson, 1964).

**FIGURE 2:**
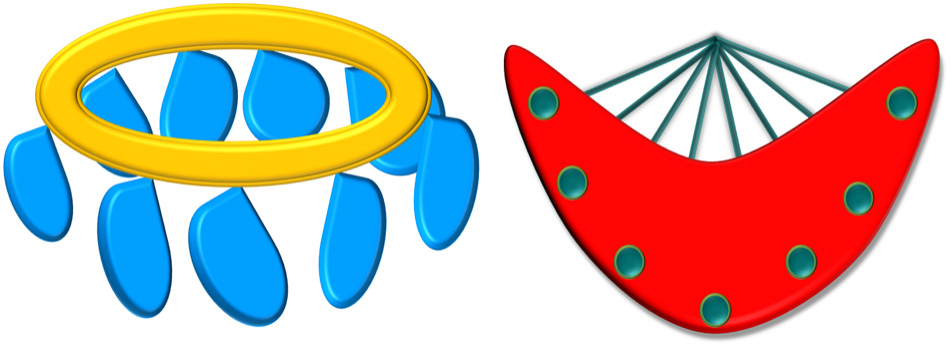
**Visual stimuli used in Experiments 1, 2, and 4**.

Acoustic analyses of the target syllable, /pa/, were conducted to ensure that the tokens matched monolingual productions within each language. The voice-onset-time (VOT) values of the three tokens ranged from 11 ms (Tone 3 production) to 18 ms (Tone 1 production). These values overlap with published VOT values of monolingual Mandarin productions that range from 11 to 18 ms (Liao, 2005; Chao et al., 2006; Chen et al., 2007; Deterding and Nolan, 2007) as well as with English monolingual values for /ba/ (Lisker and Abramson, 1967). Formant values also fell within the range of values reported for Mandarin and English monolingual productions (Mandarin monolingual F1: 1104 Hz, English monolingual F1: 850, bilingual F1: 802.7–1213.6 Hz, Mandarin monolingual F2: 1593.6 Hz, English monolingual F2: 1220, bilingual F2: 1046.3–1633.2 Hz; Peterson and Barney, 1952; Zee and Lee, 2001). F3 was not examined as it relates to lip rounding, which is not used contrastively for the target vowel in English or Mandarin. Auditory stimuli were accompanied by a visually presented novel object (see **Figure 2**) that moved in a circular path. Objects were counterbalanced to each language across participants.

English and Mandarin versions of the task were created. The target word was paired with a different object in each language. However, the target word remained the same so as to determine whether infants were capable of switching to a new set of phonological rules based on contextual cues alone.

#### Procedure

Before testing, all caregivers provided informed consent for their child’s participation, in accordance with guidelines set out by the National University of Singapore Institutional Review Board. Infants sat on their parents’ lap in a dimly lit testing suite facing a computer screen. Parents were asked not to interact with their child during the session. The experimenter observed the infant’s behavior from an adjoining room. During the experiment, both the parent and the experimenter listened to instrumental masking music.

During the task, novel objects were presented in the context of naming phrases to infants in the Switch task (Stager and Werker, 1997; Fennell and Waxman, 2010). The experiment consisted of a habituation and test phase. Before each trial, an attention getter was presented. Trials were initiated when infants oriented to the visual display. When the infant fixated to the visual display, the habituation phase commenced. Habituation consisted of repeated presentations of the target word /pa/ in Tone 3, embedded within the naming phrases and presented with the novel object. The habituation phase terminated when infant’s looking times to two trials decreased to less than 65% of two longest consecutive trials or until the infant completed a maximum of 24 trials. This habituation criterion was informed by a prior study that used the Switch task with carrier sentences (Fennell and Waxman, 2010). Once either of these criteria was met, the test phase commenced. The test phase included a Same trial and two Switch trials as adopted in previous studies (e.g., Curtin, 2010; Escudero et al., 2014). Trial order was counterbalanced across infants. For the Same trial, infants were presented with the word-object pairing to which they had habituated (i.e., /pa/ in Tone 3). The Switch trials violated this pairing, presenting infants with the same visual stimulus but paired with the target word /pa/ produced in Tones 1 and 2, respectively. Across all phases, trials lasted for a maximum of 20 s, or until the child looked away from the screen for more than 2 s. Trials were repeated if infants fixated to the screen for less than 1 s. Following the test trials, a post-test was presented. This consisted of a novel object produced by a different female speaker and labeled as a /pI/ produced in a novel tone (Tone 4). The object was animated to enlarge and shrink on the screen. A post-test trial is commonly included in the Switch paradigm to provide an indication of attention to the task during the terminal phase of the experiment. In prior studies (e.g., Fennell et al., 2007; Byers-Heinlein et al., 2013), fixation to the last habituation block has been compared to fixation to the post-test trial. Elevated attention (recovery) between these is recruited as an interpretative safeguard against a Type II error: in the event of a null result whereby fixation to Same and Switch trials do not differ, the presence of recovery between the last habituation block and the post-test trial indicates that this is unlikely to be accounted for by fatigue or disengagement from the experiment during the test trials. An example of the stimuli is provided in **Figure 2**. Infants were presented with a Mandarin and an English version of the same task. The order of presentation of the English and Mandarin task was counterbalanced across infants. Between the two tasks, infants were presented with a 1-min non-verbal cartoon.

Both the Switch task and preferential looking approaches are well-established measures of infants’ sensitivity to phonological variation when learning new words. However, in pilot studies, a preferential looking approach to the present task (including relevant parameters such as two languages, three test trial types) proved excessively demanding for participants. Each session was substantially longer than the auditory word segmentation task used within subjects by Singh and Foong (2012), and in recent research, a preferential looking approach to the question of perceptual switching was only successfully used in older children at 3–5 years of age (see Singh and Quam, 2016). As a consequence, the Switch task was selected for the current study. It should be noted that it is possible to use the Switch task to measure sensitivity to phonological variation using two objects (e.g., Werker et al., 2002; Fennell and Werker, 2003). However, familiarization with two objects could not be integrated into a design with a three trial [Same; Switch (distinct); Switch (similar)] test phase. An alternative design would have been to incorporate a two-trial (Same and Switch) test phase and manipulate contrast salience across participants. We prioritized the manipulation of salience as a within-subjects contrast in light of the fact that our sample comprised bilingual infants; a between-subjects comparison between two groups of bilinguals can introduce differences in performance due to background variables (specifically, the nature and extent of bilingual input, which are hard to match across bilingual groups with precision). Uncontrolled effects of error variance due to individual variation are somewhat mitigated by within-subjects comparisons, which motivated our decision to incorporate a single object and to manipulate salience within participants for each experiment. Although less common than a two-object paradigm, a single-object Switch paradigm has been used in several prior studies (see Stager and Werker, 1997; Werker et al., 1998; Pater et al., 2004; Thiessen, 2007; Fennell and Waxman, 2010; Fennell, 2012).

### Results

All infants habituated within the 24 trial maximum habituation window. A preliminary analysis was conducted to determine whether participants recovered to the post-test by comparing the last habituation block to the post-test stimulus. A 2 × 2 (phase: last habituation block/post-test × language: English/Mandarin) repeated-measures ANOVA revealed a main effect of phase [*F*(1,34) = 13.91, *p* = 0.001, $\eta_{p}^{2}$: 0.29], accounted for by an elevation in fixation times between the last habituation block and the post-test. There were no effects of language on fixation times nor was there an interaction of phase and language on fixation times (*p* > 0.8).

An initial set of analyses was conducted to determine if there was an effect of test order on fixation times to test trials. A 3 × 2 × 2 (Trial type: Same; Switch-similar; Switch-distinct × Language: English; Mandarin × Order: Mandarin first; English first) repeated-measures ANOVA was conducted with fixation times during test trials as the dependent variable. Results revealed no effects of interactions with order (*p* > 0.3). Fixation times were therefore collapsed across test orders for subsequent analyses.

**FIGURE 3:**
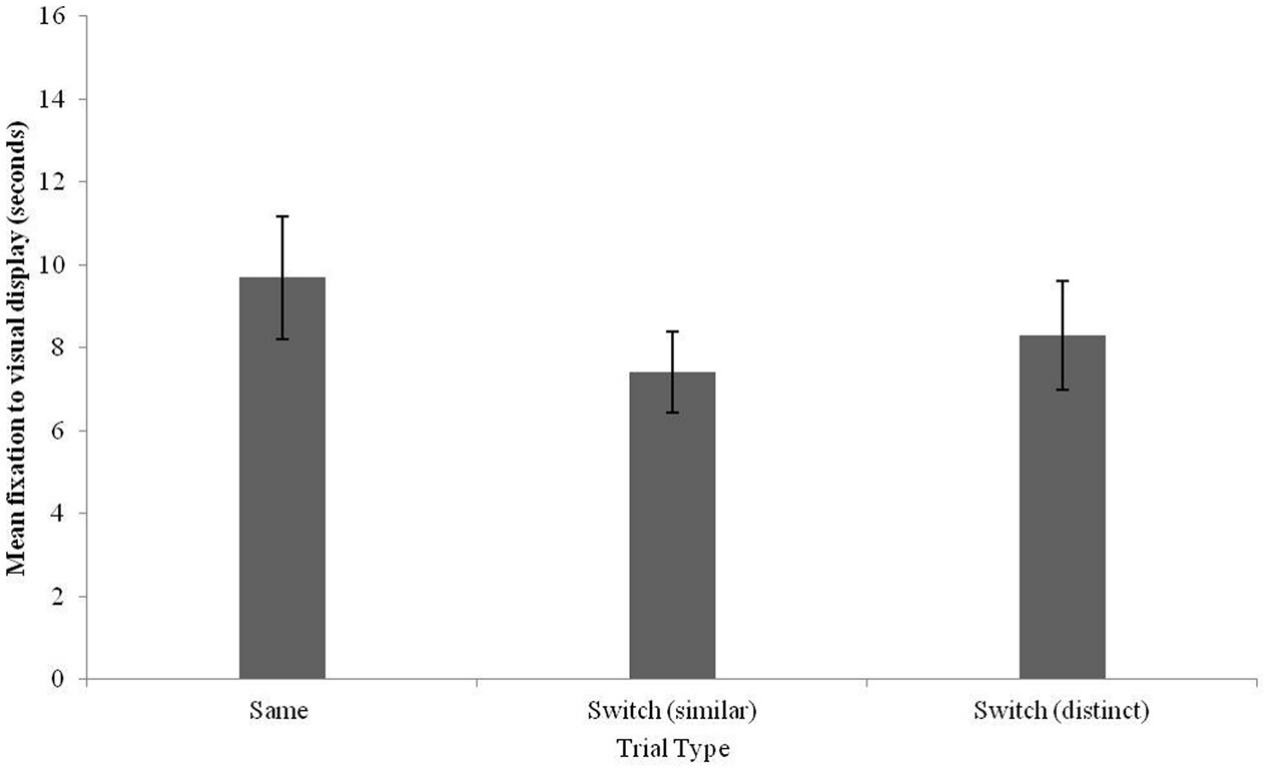
**Fixation times to the visual stimulus for Same, Switch (similar), and Switch (distinct) trials in 12–13-month-old bilingual infants (error bars: SEM)**.

As the other of test trials was rotated across participants, a preliminary analysis was conducted to investigate effects and interactions test trial order, trial type, and language, revealing no effects or interactions with test trial order (*p* > 0.6). Test trial order was excluded from subsequent analyses. A 3 × 2 Trial type × Language repeated-measures ANOVA was then conducted. Results revealed a main effect of trial type [*F*(2,34) = 11.18, *p* = 0.0001, $\eta_{p}^{2}$ = 0.39], no main effect of language (*p* = 0.23) and no interaction of trial type and language [*F*(2,34) = 2.46, *p* = 0.1]. Planned comparisons were conducted within each language to determine whether participants differed in how they responded to each tone change based on the language of testing. For each language, a repeated measures ANOVA was conducted to determine the effect of trial type (Same; Switch-similar; Switch-distinct) on fixation times to test trials. When participants were tested in Mandarin, results revealed a main effect of trial type [*F*(2,34) = 10.56, *p* = 0.0001m, $\eta_{p}^{2}$: 0.39]. Simple contrasts revealed higher fixation times to Switch-distinct trials than to Same trials [*F*(1,17) = 20.35, *p* > 0.0001, $\eta_{p}^{2}$: 0.54] as well as higher fixation times to Switch-similar trials than to Same trials [*F*(1,17) = 5.93, *p* = 0.03, $\eta_{p}^{2}$: 0.26]. A *post hoc* analysis comparing fixation times to Same and Switch trials for the two Switch trials (similar and distinct) demonstrated that differences in Same–Switch trials were greater for when the Switch involved a distinct contrast (i.e. change from Tone 3 to Tone 1) than when it involved a similar contrast [i.e., change from Tone 3 to Tone 2; *t*(17) = 2.3, *p* = 0.04 (Cohen’s *d*: 0.57)]. This analysis revealed effects of perceptual salience on tone integration in Mandarin, although both similar and distinct substitutions were recognized as lexically contrastive. When participants were tested in English, results revealed a main effect of trial type [*F*(2,34) = 3.27, *p* = 0.05]. Simple contrasts revealed no significant difference in fixation to Switch-distinct trials than to same trials [*F*(1,17) = 3.15, *p* = 0.1] nor to Switch-similar trials than to Same trials [*F*(1,17) = 0.54, *p* = 0.47]. Fixation times to each trial type for English and Mandarin are plotted in **Figure 3**.

Findings suggest that bilingual English–Mandarin infants recognized the lexical relevance of tone in English and Mandarin, responding differentially to tone variants based on the language in which words were introduced. In a second experiment, Mandarin monolingual infants were tested recognition of tone-matched and tone-varying words in the same task as employed in Experiment 1 (Mandarin version). The goal of this experiment was to provide a monolingual point of comparison for findings obtained in Experiment 1. Given that bilingual infants were sensitive to tone variation when words were introduced in Mandarin, it was expected that Mandarin monolingual infants would be comparably sensitive to tone variation.

## Experiment 2

We investigated Mandarin monolingual infants’ sensitivity to tone changes in a similar paradigm as that used in Experiment 1. The primary methodological difference with Experiment 1 was that all participants were tested in Mandarin only. As in Experiment 1, tone changes consisted of similar and distinct contrasts.

### Method

#### Participants

Our sample comprised 18 12- to 13-month-old Mandarin monolingual infants (age range: 12 months 11 days to 13 months 13 days, average = 12 months 24 days). All infants were full-term births with no known developmental delays or disabilities. Data from two additional infants were excluded due to failure to complete the testing session. All infants had more than 90% exposure to Mandarin as measured by the Language Exposure Questionnaire (Bosch and Sebastián-Gallés, 1997).

#### Stimuli

Auditory and visual stimuli for the Mandarin testing session were identical to Experiment 1 (Mandarin version).

#### Procedure

The experimental procedure and all other experimental parameters were identical to the Mandarin version of Experiment 1.

### Results

All infants habituated within the 24 trial maximum habituation window. The number of trials to habituation and the total habituation time for each experiment is reported in **Table 2**. As in previous studies (see Fennell et al., 2007; Byers-Heinlein et al., 2013), a preliminary analysis was conducted to determine whether participants recovered to the post-test by comparing the last habituation block to the post-test stimulus. A paired samples *t*-test revealed a significant elevation in fixation times between the last habituation block and the post-test [*t*(17) = 2.57, *p* = 0.02].

**Table 2:** Summary of habituation measures.

	**Trials to habituation: mean (*SD*)**		**Total habituation time (seconds): mean (*SD*)**	
	
	Mandarin session	English session	Mandarin session	English session

Experiment 1	6.44 (2.66)	6.56 (2.43)	86.36 (51.53)	70.16 (47.39)
Experiment 2	6.5 (3)		79.01(53.54)	
Experiment 3	7.27 (2.86)		67.61 (30.61)	
Experiment 4	6.05 (2.34)		76.71 (46.24)	

**FIGURE 4:**
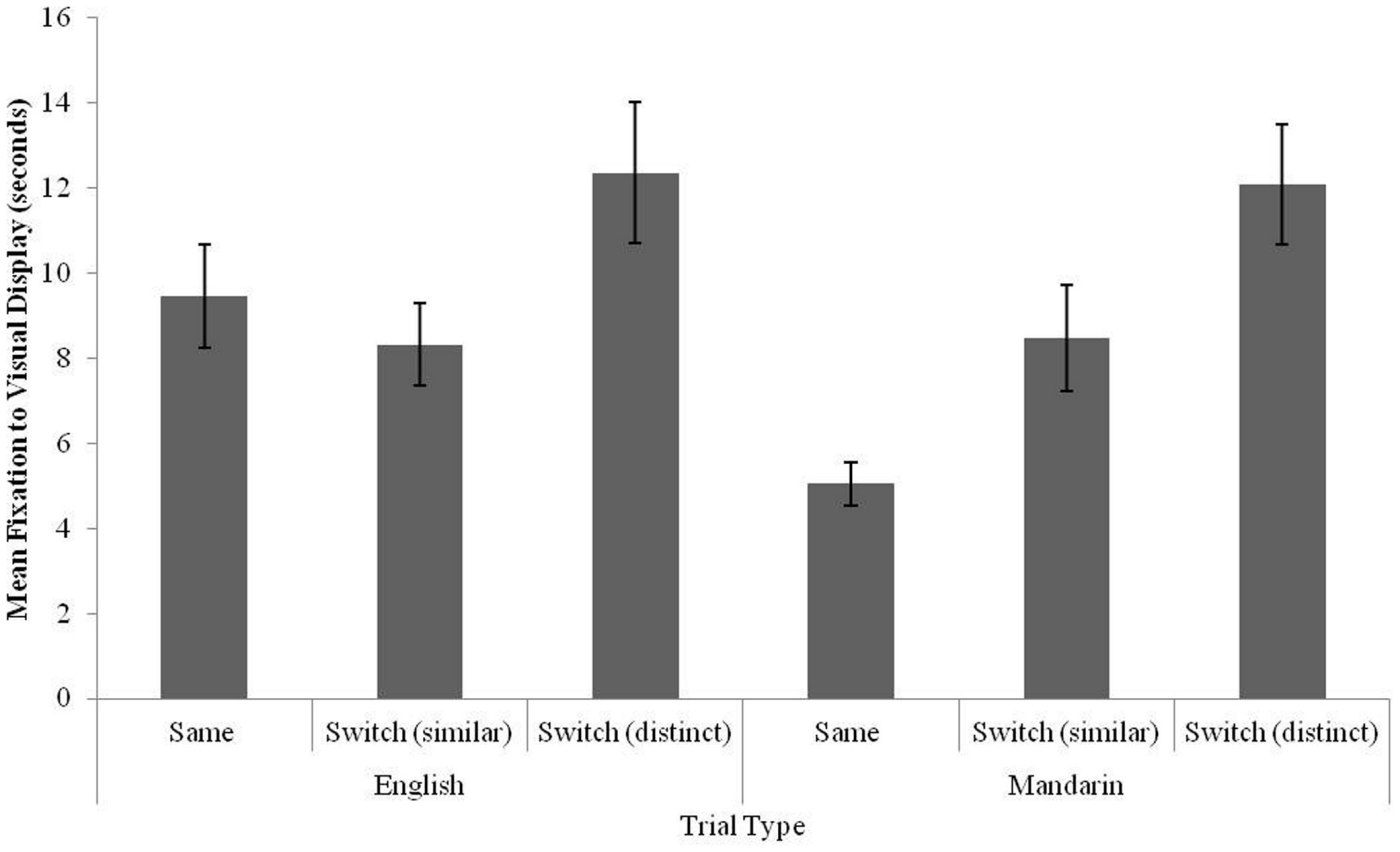
**Fixation times to the visual stimulus for Same, Switch (similar), and Switch (distinct) trials in 12–13-month-old Mandarin monolingual infants (error bars: SEM)**.

Fixation times were logged for Same trials, Switch (similar) and Switch (distinct) trials. These values are plotted in **Figure 4**. A preliminary analysis conducted with test trial order as a between-subjects factor and trial type as a within-subjects factor revealed no effects or interactions with test trial order (*p* > 0.6). A one-way repeated-measures ANOVA with test trial as the within-subjects factor revealed no effect of trial type [*F*(2,34) = 1.31, *p* = 0.28]. A comparison of fixation times to Same trials as compared to each Switch trial revealed no difference in fixation times to Same versus Switch (similar) trials [*t*(17) = 0.89, *p* = 0.39] or between Same and Switch (distinct) trials [*t*(17) = 1.69, *p* = 0.11].

In comparing the results of Experiments 1 and 2, it is striking that infants with monolingual exposure to Mandarin did not differentiate tones when learning a novel word whereas those learning English and Mandarin did demonstrate sensitivity to tone when listening to Mandarin. It is possible that bilingual infants’ integration of tone in Mandarin was related to having had prior exposure to the object label during the English session. If this were the case, one would predict effects of the order of testing on performance in the Mandarin session. As half of the infants underwent an English testing session first and half underwent a Mandarin testing session first, a 3 × 2 [test trial (same; switch (similar); switch (distinct)) × order (English first; Mandarin first)] mixed ANOVA was conducted with fixation times to the Mandarin test trials as a dependent variable. Results revealed a main effect of trial type [*F*(2,32) = 9.97, *p* = 0.0001, $\eta_{p}^{2}$ = 0.38], no effect of order of testing [*F*(1,16) = 1.2, *p* = 0.28] and no interaction of test order and trial type [*F*(2,32) = 0.7, *p* = 0.94].

A secondary set of analyses was performed on habituation data in order to determine whether monolingual and bilingual infants were distinguished by their habituation profiles. A one-way ANOVA was conducted to compare the total time accrued during habituation and on the number of trials to habituation between Mandarin monolinguals, English–Mandarin bilinguals (Mandarin session) and English–Mandarin bilinguals (English session). There was no effect of group on total time accrued during habituation [*F*(2,53) = 0.18, *p* = 0.84]. Likewise, there was no effect of group on the number of trials to habituation [*F*(2,53) = 0.69, *p* = 0.51]. These analyses suggest that the profile of stimulus encoding did not differ across groups.

The present results suggest that Mandarin monolingual infants were not sensitive to labels for a familiarized object that had undergone a tone substitution, whether the substitution was due to a shift to a similar or distinct tone. This was unexpected given findings from non-tone language learning infants demonstrating that infants at 14 and 17–18 months of age were sensitive to lexical tone distinctions when learning new words (Hay et al., 2015; Singh et al., 2015). Differences between experiments will be revisited in the Section “Discussion.” Using a different paradigm, Experiment 3 sought to determine whether Mandarin learning monolingual infants could discriminate the lexical tones that they were not able to integrate in Experiment 1. Given that tone learning infants have been shown to discriminate lexical tones at 4, 6, and 9 months of age (Mattock and Burnham, 2006; Yeung et al., 2013), it was hypothesized that Mandarin learning infants would discriminate Mandarin tones at 12–13 months.

## Experiment 3

In this experiment, Mandarin monolinguals were tested on their ability to discriminate Tone 3 from Tones 1 and 2 in a phoneme discrimination paradigm. Participants were habituated to Tone 3 and then presented with an alternating string of Tone 3 and a contrastive tone (Tone 1 or 2). They were then re-exposed to Tone 3 and presented a second alternating string of Tone 3 and the other contrastive tone (Tone 1 or 2).

### Method

#### Participants

Our sample comprised eighteen 12- to 13-month-old infants who had been monolingually exposed to Mandarin (age range: 12 months 11 days–13 months 22 days, average = 12 months 24 days). Data from two additional infants was excluded as testing was incomplete due to fussiness. The language criteria used for this study was identical to that of Experiment 1.

#### Stimuli

Auditory stimuli consisted of the syllable /pa/, recorded in Mandarin Tones 1, 2, and 3. Multiple tokens were recorded, and four tokens of each tone were selected for the final stimuli. The VOT values and pitch contours for these syllables are equivalent to those described in Experiment 1. Stimuli were concatenated to form three trial types: (1) a Control trial, which featured only Tone 3 tokens, (2) an Alternating distinct tone pair trial, which had alternating tokens of Tones 1 and 3, and (3) an Alternating similar tone pair trial, which consisted of alternating tokens of Tones 2 and 3. All strings were 30 s long, and were created by repeating the stimuli systematically, with an interstimulus interval of 1 s. All strings were also paired with the visual stimulus of a stationary red-and-black checkerboard pattern presented against a white background.

#### Procedure

As with the previous experiments, testing was conducted in a quiet, dimly lit room, where the infant sat in their caregiver’s lap, facing a computer screen. The experimenter observed the infants’ responses via a CCTV system from an adjoining room. Both the experimenter and parent listened to instrumental music at a volume that masked the stimuli.

The procedure used was an adapted version of the stimulus alternating paradigm developed and previously used to assess discrimination of two contrasts within the same infant (Tyler et al., 2014; see Best and Jones, 1998; Maye et al., 2002; Mattock et al., 2008 for additional demonstrations of the paradigm). Infants were first presented with the attention getter. At the first fixation to the visual display, the habituation phase commenced. In the habituation phase, infants were presented continuous tokens of Tone 3. Trials lasted for a maximum of 30 s, or until the infant looked away from the screen for more than 2 s. At the end of each trial, the attention getter was presented again. The habituation phase continued until the infant’s looking time to the final three consecutive trials decreased to less than 50% of the total look time to the first three consecutive trials, or until the infant completed a maximum of 20 trials. This habituation criterion was informed by previous investigations of tone discrimination in Mandarin monolingual infants (see Gao et al., 2011). Once either of these criteria was met, the test phase was initiated.

**FIGURE 5:**
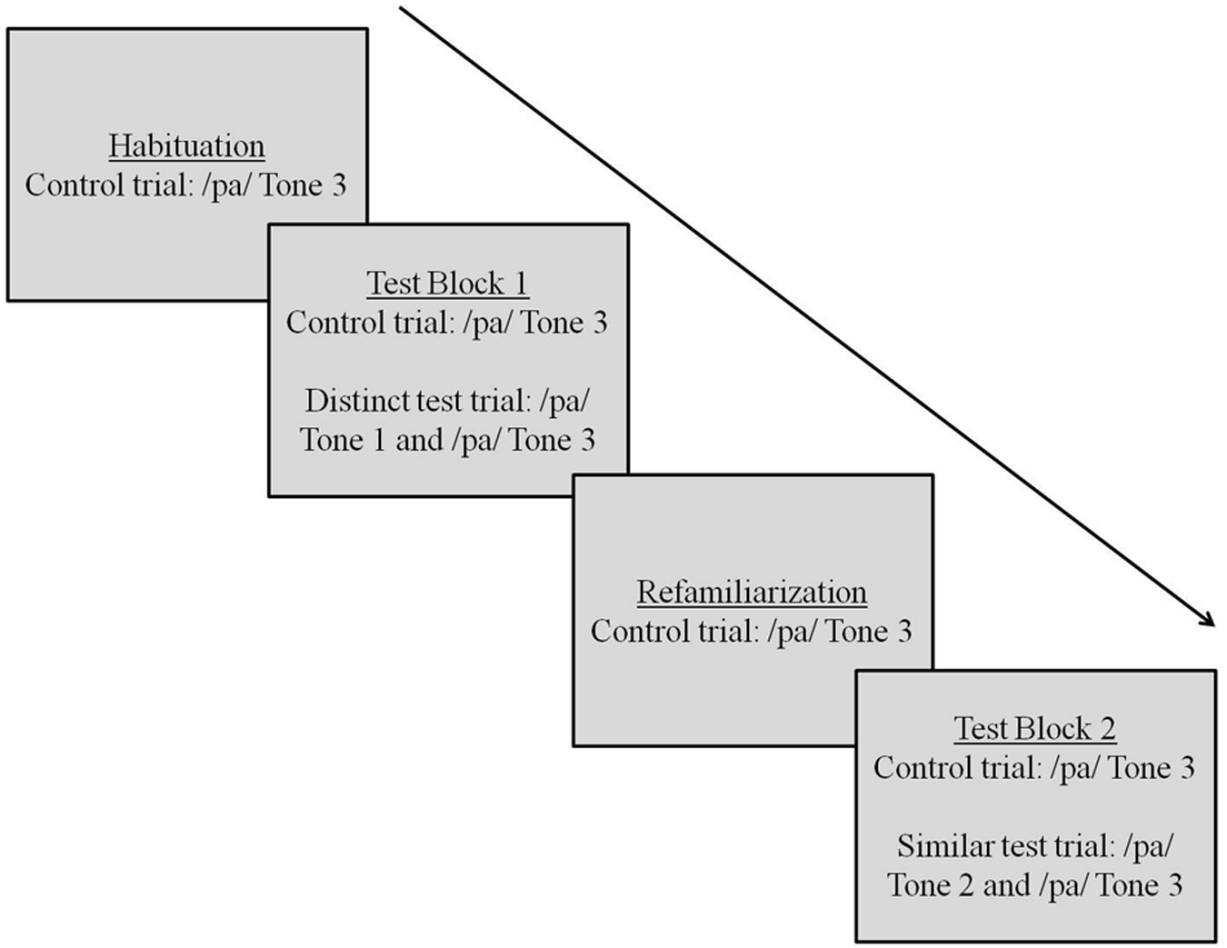
**Trial sequence for Experiment 2**.

The test phase consisted of three blocks. In the first test block, infants were first presented a Control trial (repetitions of Tone 3). This was followed by a Test trial, consisting of alternations of Tones 2 and 3 (similar) or of Tones 1 and 3 (distinct). Infants were then presented with three trials, each containing repetitions of Tone 3. The purpose of this phase was to reinstate Tone 3 as the basis for further comparisons with a contrastive tone. Following this, infants were presented with a second test block, comprising a Control trial (repetitions of Tone 3) and a second Alternating trial consisting of tonal alternations that had not been previously presented (either Tones 1 and 3 or Tones 2 and 3). The trial sequence for this experiment is depicted in **Figure 5**. The order of presentation of test blocks was counterbalanced across all infants, such that half the infants were presented with the distinct tone pair in the first alternating test trial and half were presented with the similar tone pair in the first alternating test trial.

### Results

All infants habituated within the 20 trial maximum habituation window. Difference scores were calculated for each infant, by subtracting the fixation times for each Control trial from the Alternating trial that followed it. Thus, infants each had two difference scores: one reflected dishabituation to the alternating trial consisting of a similar tone contrast (Tones 2 and 3) and one reflected dishabituation to the alternating trial consisting of the distinct tone contrast (Tones 1 and 3). A difference in fixation to the checkerboard display between Control and alternating blocks that deviates significantly from zero indicates that infants discriminated the Control tone from the tone presented in the alternating trial.

**FIGURE 6:**
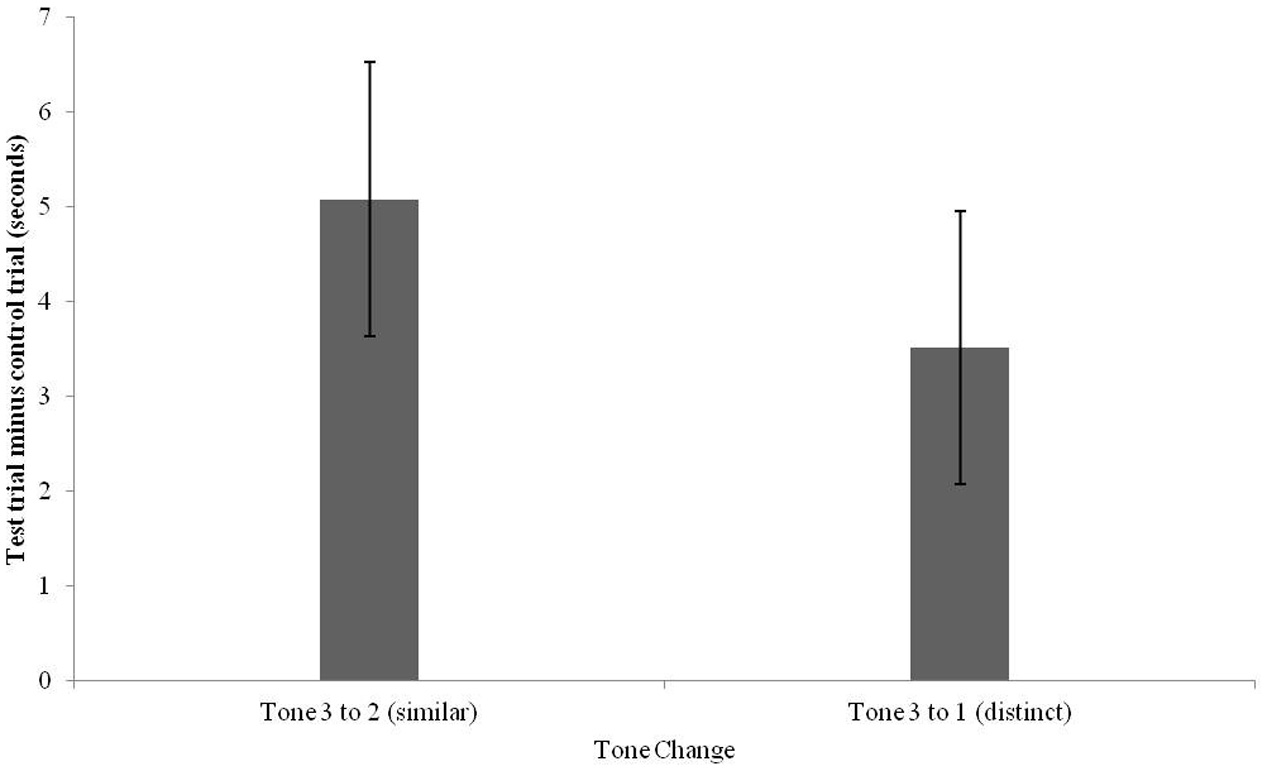
**Mean differences in looking time to control (non-alternating) versus test (alternating) trials for distinct (Tone 3 to Tone 1) and similar (Tone 3 to Tone 2) changes (error bars: SEM)**.

A 2 × 2 repeated measures ANOVA (Contrast: similar vs. distinct) × 2 (Order: similar first vs. distinct first) was computed with difference scores as the dependent variables. No effects of order were found (*p* > 0.3) and thus order of presentation was excluded from subsequent analyses. To examine whether infants successfully discriminated each contrast, one-sample *t*-tests were used to analyze infants’ difference scores in relation to baseline. This analysis revealed that infants’ difference scores for the distinct contrast were significantly greater than zero, *t*(17) = 3.31, *p* = 0.003, Cohen’s *d* = 1.17. Similarly, difference scores for the similar contrast (*M* = 3.79, *SD* = 6.45) were also significantly greater than zero, *t*(16) = 2.44, *p* = 0.03, Cohen’s *d* = 0.81. Difference scores for these contrasts are depicted in **Figure 6**.

These results suggest that at 12–13 months, Mandarin monolinguals were sensitive to the same tone contrasts introduced in Experiment 2. Thus, while the Mandarin monolinguals successfully discriminated these contrasts, they appeared unable to integrate this information when learning new words. This conclusion should be qualified by the fact that a different paradigm was used to track auditory sensitivity to tone versus integration of tone when learning novel words. Hence, we do not conclude from this study that when presented with equivalent task demands in referential versus non-referential context, infants are sensitive to lexical tone only in the latter case. Rather, our claim is that in particular tasks known to elicit auditory sensitivity to tone contrasts, such as the Stimulus Alternating Paradigm, infants are indeed sensitive to the distinction between Tones 1 and 3 and Tones 2 and 3. Prior studies (e.g., Stager and Werker, 1997) that have tracked sensitivity to a single contrast have measured sensitivity in discrimination and word learning by using highly similar paradigms, replacing the object to be learned with a checkerboard. In our study, on account of simultaneously tracking sensitivity to two contrasts within the same participant and within a single experimental session, we opted for an equally well-established paradigm to measure phoneme discrimination. This paradigm allowed us to maintain some of the elemental components of the word-learning paradigm used in Experiment 2, specifically measurement of sensitivity to two contrasts within a single session and infant. It should also be noted that our findings from Experiment 3 are consistent with prior research using alternative discrimination paradigms that also demonstrate lexical tone discrimination in tone learning infants between 9 and 12 months of age (see Mattock and Burnham, 2006; Tsao, 2008; Yeung et al., 2013).

In light of the finding that Mandarin learning infants appeared to discriminate words based on tone, yet did not integrate these tones into newly learned words (albeit in a paradigm with different experimental parameters), Experiment 4 was designed to investigate whether older Mandarin monolingual infants could integrate tones into newly learned words. Infants undergo significant change in their abilities to learn similar sounding words by 17–18 months (e.g., Stager and Werker, 1997) and specifically, non-tone language learning infants mature in their language-specific integration of tone between 14 and 17 months (Hay et al., 2015). It is possible that tone language learners also mature in this capacity as they approach 18 months and construe tones as a source of contrast when learning new words. It was therefore hypothesized that by 18 months, tone-learning infants would differentiate newly learned words based on tones.

## Experiment 4

To determine whether older Mandarin monolinguals would be successful at detecting tone changes in a word-object association task, we tested 17- to 18-month-old Mandarin monolingual infants on the same procedure as Experiment 2.

### Method

#### Participants

The sample comprised eighteen 17- to 18-month-old monolingual Mandarin infants (age range: 17 months 3 days to 18 months 29 days, average age = 17 months 21 days). Four additional infants were tested but excluded due to experimental error (*n* = 2), fussiness (*n* = 1) or on account of data that deviated from the group mean by more than 3 standard deviations (*n* = 1).

#### Stimuli

Stimuli were identical to those used in Experiment 2.

#### Procedure

The procedure was identical to that of Experiment 2.

### Results

All infants habituated within the 24 trial maximum habituation window. A comparison of the last habituation block and post-test trials revealed a significant increase in fixation to the post-test trial, *t*(17) = 4.1, *p* = 0.001. A preliminary analysis conducted with test trial order as a between-subjects factor and trial type as a within-subjects factor revealed no effects or interactions with test trial order (*p* > 0.6).

**FIGURE 7:**
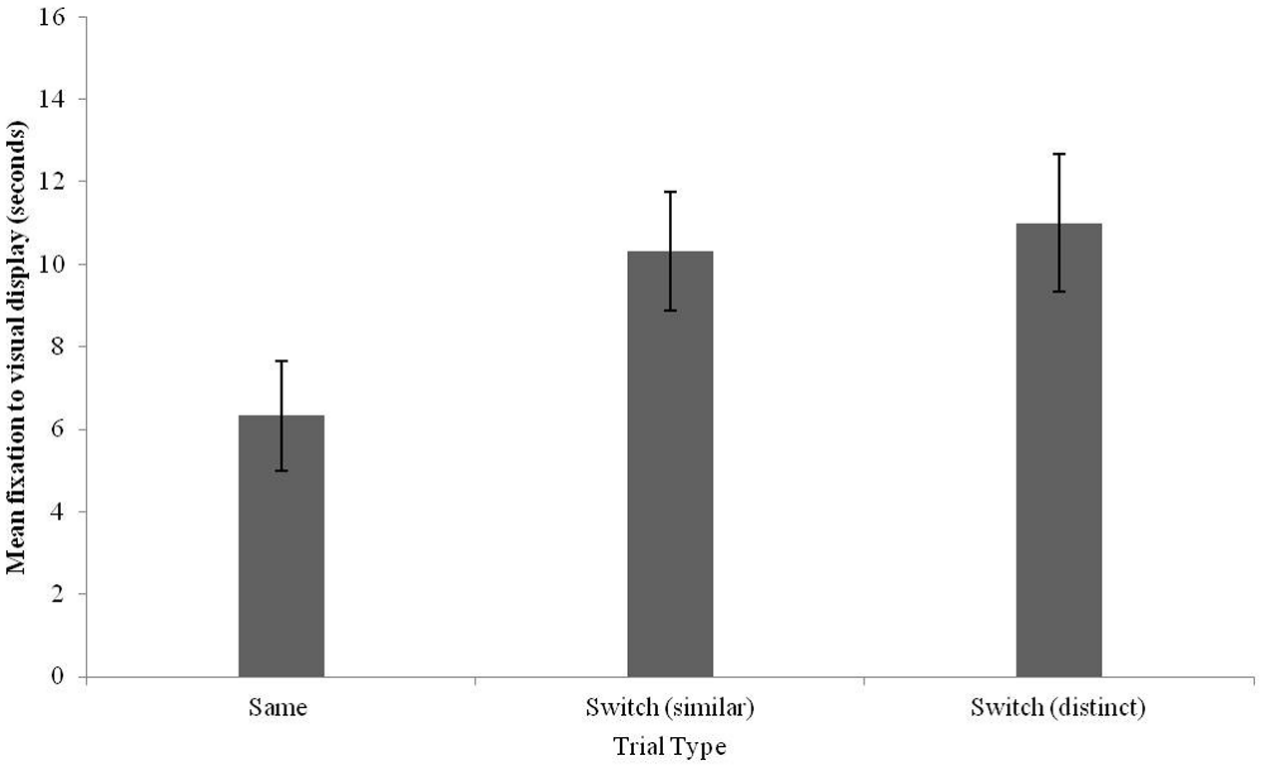
**Fixation times to the visual stimulus for Same, Switch (similar), and Switch (distinct) trials in 17–18-month-old Mandarin monolingual infants (error bars: SEM)**.

Further analyses focused on Same–Switch differences for each type of Switch trial (similar and distinct). A repeated-measures ANOVA was conducted to compare fixation times to each trial type [Same, Switch (similar) and Switch (distinct) tones], revealing a main effect of trial type [*F*(2,34) = 5.63, *p* = 0.008, $\eta_{p}^{2}$ = 0.25]. Planned contrasts revealed an increase in fixation to tone shifts that were both similar [*F*(1,17) = 6.53, *p* = 0.02, $\eta_{p}^{2}$ = 0.28] and distinct [*F*(1,17) = 15.36, *p* = 0.001, $\eta_{p}^{2}$ = 0.48]. These results are graphed in **Figure 7**. The results from Experiment 4 demonstrate that by 17–18 months, Mandarin learning infants are sensitive to similar and distinct tone variation when learning new words.

## Discussion

The present set of studies was designed to investigate the extent to which lexical tone is phonologically articulated within the bilingual and monolingual infant lexicon. Infants’ sensitivity to lexical tone was examined across four experiments. Experiment 1 investigated bilingual English–Mandarin infants’ sensitivity to lexical tone variation in each of their native languages. Infants exhibited language-selective integration of lexical tone at this stage, contrasting newly learned words by tone variation in a Mandarin context and disregarding tone variation in an English context even though they were tested in each language in immediate succession. In this experiment, there were effects of perceptual salience of the tone contrast on infants’ sensitivity to tone variation in Mandarin. However, these effects were secondary in that they did not eclipse infants’ overall recognition of the lexical functions fulfilled by pitch in Mandarin. In Experiment 2, we investigated 12- to 13-month-old monolingual Mandarin learning infants’ abilities to integrate lexical tone into memories of newly learned words. Infants demonstrated a relative insensitivity to tone variation, irrespective of whether the variation was introduced by a similar or distinct mispronunciation. In Experiment 3, Mandarin monolingual infants were tested on their ability to discriminate the lexical tones presented in Experiment 1, revealing that both similar and distinct tone pairings were robustly discriminated in a habituation paradigm between 12 and 13 months. Finally, in Experiment 4, Mandarin learning monolingual infants were tested on the same paradigm as Experiment 2 at an older age (17- to 18-months), demonstrating an ability to integrate lexical tone variation into newly learned words and to detect similar and distinct mispronunciations in equal measure.

Previous investigations of infants’ abilities to learn similar sounding words have focused on their sensitivity to segmental detail, most notably, to the onset consonant of a word (e.g., “bih” versus “dih”) (e.g., Stager and Werker, 1997; Pater et al., 2004; Fennell et al., 2007; Fennell and Byers-Heinlein, 2014, but see Curtin et al., 2009). In the aggregate, these findings suggest that monolingual infants are not able to learn similar sounding words differing by onset consonant until 17 months (Stager and Werker, 1997, but see MacKenzie et al., 2011), although this ability has been shown to emerge at 14 months when infants received contextual support (Fennell and Waxman, 2010). Our findings with Mandarin monolingual infants suggest that even with contextual support (i.e., naming phrases) infants were not able to map tonal variants onto different objects at 12–13 months and were only able to do so at 17–18 months. Given that the majority of prior studies investigating mastery of similar sounding words has been conducted with infants 14 months of age and older, it is difficult to compare the course of acquisition of segmental contrasts versus tone contrasts based on the present study. In contrast to Mandarin monolingual infants, the most surprising finding to emerge from the current set of studies is that bilingual infants demonstrated precocity in their ability to integrate tone variation in a language-selective manner as early as 12–13 months. Unlike monolingual infants, they were able to integrate variation in lexical tone in a Mandarin context. Within the same laboratory session, when presented with a new word-object pairing in English naming phrases, they were able to disregard the same sources of variation when tested in English. This finding is somewhat unexpected given the task demands faced by bilinguals in this study whereby they would have had to inhibit the phonological rules of one of their native languages in each task. The experiment was designed such that the phonetic properties of the target words remained the same across languages, suggesting that context alone may have enabled a language-specific integration of tone. In prior research, bilingual and monolingual infants have been shown to be similar to one another – assuming they receive input commensurate with their language environment – in learning similar sounding words with no clear evidence of a bilingual advantage (Mattock et al., 2010; Byers-Heinlein and Fennell, 2014). However, our study deviates from prior studies in this area in that previous research has focused exclusively on how bilinguals negotiate sound contrasts that distinguish meaning on both of their languages. In contrast, the current study investigates sensitivity to a source of phonological variation that categorically conflicts across languages (i.e., it is phonemic in one and non-phonemic in the other). Three possible reasons for a bilingual advantage in this task are discussed in turn.

First, it should be noted that tone does not only introduce phonological conflict for bilingual learners. Monolingual Mandarin learners also confront potential conflict within their native language on account of tone. Pitch movements drive lexical changes in tone but they also drive changes in intonation that are non-lexical in Mandarin. A learner of Mandarin therefore has to selectively integrate pitch variation that corresponds to lexical tone categories when learning new words and to disregard that which distinguishes intonational contrast when defining words. The challenge inherent in this duality is evidenced by findings that even adult speakers of Mandarin Chinese are sensitive to tone-intonation conflict in native sentence processing (e.g., Yuan and Shih, 2004). Therefore, tone introduces intrinsic conflict for monolingual Mandarin learners as well as for bilingual learners. It is possible that bilingual infants are better able to negotiate this conflict on account of collateral cognitive changes that are thought to arise from bilingual experience. This possibility derives from a broad swath of research demonstrating a bilingual advantage in negotiating conflicting information both in linguistic and non-linguistic tasks (e.g., Bialystok et al., 2004, 2008; Costa et al., 2008; Kovács, 2009). The presence of conflict in the task may have harnessed bilinguals’ extant advantages for cognitive control in the face of conflict, an advantage apparent in infancy (Kovács and Mehler, 2009a). As such, it is possible that cognitive control advantages conferred upon the bilingual infant permeate early language processing, aiding in the de-activation of the phonological structure of the one language when processing the other.

An explanation predicated on a bilingual advantage in conflict resolution presupposes that the advantage demonstrated in word learning is secondary to a general cognitive advantage to emerge from bilingual exposure. However, a second possibility is that the bilingual advantage observed herein is specific to language. Prior studies with bilingual children and adults have revealed a bilingual advantage in mastering the rules of the native languages, often characterized as a metalinguistic benefit of bilingualism (e.g., Bialystok, 1988; Bialystok et al., 2003). Although these studies have focused largely on mastery of the grammatical systems of each language, metalinguistic advantages appear to transcend grammatical knowledge and extend to mastery of the sound system (Campbell and Sais, 1995). A mechanism commonly advanced for why bilingualism may promote metalinguistic awareness may provide a second potential explanation for our findings. The mere presence of conflict – or structural differences – across languages may highlight relevant properties of each language to bilingual learners (Bialystok and Hakuta, 1994; Friesen and Bialystok, 2012). Although rhetorically, researchers have appealed to cross-language conflict as a basis for metalinguistic advantages (see Bialystok and Hakuta, 1994), tests of metalinguistic awareness in bilinguals have not generally measured sensitivities to linguistic cues that functionally conflict across the two languages of a bilingual. The normative approach has been to measure sensitivity to the rules of one language (see Bialystok, 2001, for a review). The current study suggests that mastering properties of languages that conflict, which intuitively should be more complex to negotiate, may be consolidated earlier in bilinguals. It is therefore possible that the precocity observed among bilingual infants in the present study derives directly from experience with conflicting linguistic rules. In other words, noticing that pitch cues effect referential change in one language but not in the other may facilitate an awareness of pitch as a relevant – and contrastive – feature of language to young learners.

Finally, the advantage observed in bilingual infants may derive from a specific sensitivity to pitch. Prior research demonstrates that bilinguals are more sensitive to prosody and more generally, to the encoding of pitch in comparison to monolinguals in both infancy and adulthood (Krizman et al., 2012; Gervain and Werker, 2013). In comparison with monolingual infants, bilingual infants more readily incorporate pitch movements as a cue to linguistic structure even if they are not learning a tone language (Gervain and Werker, 2013). It is possible that that the bilingual advantage observed in the present study may be limited to the specific source of variation contained within this study – vocal pitch. Further research could test this hypothesis by investigating sensitivity to segmental phonological conflict across languages in monolingual and bilingual learners.

In addition to demonstrating bilingual infants’ facility with negotiating phonological conflict, a second contribution of the present study is to chart tone sensitivity in native learners of a tone language. From our findings, it appears that native tone language learners do not incorporate tone into newly learned words until 18 months. At 12–13 months, Mandarin learning infants appear insensitive to tone variation in newly learned words, an effect that does not reflect a limitation in discriminating the tone pairs used in this study but rather a specific limitation in integrating tones into novel word-object mappings. A disconnect between the capacity for auditory discrimination of native contrasts and integration of these contrasts into names for objects has been reported with regards to consonant variation (see Stager and Werker, 1997). However, this disconnect, often termed the word learning ‘paradox,’ is often alleviated when words are embedded in naming phrases that highlight the referential nature of the task at 14 months (Fennell and Waxman, 2010). In the present study, however, even when supported with naming phrases, 12- to 13-month-old monolingual Mandarin learning infants were not able to integrate tone variation into newly learned words. It is possible that the ability to profit from naming phrases develops closer to 14 months and was therefore not captured within the time frame under investigation in the present study. However, it is also possible that tone variation effected by pitch movements is more challenging to bind to the lexicon than segmental variation. Pitch serves a broad range of functions in all languages and tone languages are no exception. In Mandarin Chinese, pitch cues make important non-lexical distinctions, such as distinguishing questions versus statements (Yuan et al., 2002), contrastive prosodic stress (Xu, 1999), as well as contrasting vocal emotions (Li et al., 2011). The functional differentiation of pitch may be a complex process for tone language learner and this complexity may prolong the process of assigning distinct communicative functions to pitch variation. One source of support for this comes from prior developmental research demonstrating that pitch cues to tone and intonation are only robustly dissociated as late as 4–5 years of age in Mandarin learning children (Singh and Chee, 2016). Although bilingual infants contend with the same complexity with regards to pitch, or arguably even more, enhancements in cognitive control and/or metalinguistic awareness and/or enhanced pitch sensitivities may offset the effects of this complexity. Moreover, the mere presence of conflict across languages, often thought to underlie bilingual advantages in metalinguistic awareness, may facilitate phonological integration in bilingual infants.

The finding that Mandarin learning infants did not incorporate lexical tone into newly learned words at 12–13 months is somewhat surprising in light of prior studies demonstrating that other populations associate newly learned words with tones. Integration of tones in non-tone language learning infants was evidenced at 14 and 18 months (Graf Estes and Hay, 2015; Hay et al., 2015; Singh et al., 2015) and in Mandarin–English bilinguals at 18 months (Singh et al., 2015), although it should be noted that none of these studies sampled Mandarin monolingual infants. Four possible explanations are offered for why Mandarin monolingual infants may have exhibited a different response to other language groups, such as English monolingual infants. First, as mentioned earlier, it is possible that the functional differentiation of pitch for a Mandarin learner is associated with a more complex learning pathway on account of the multiplexing of pitch in tone languages (e.g., pitch is used to contrast emotions, stress, communicative intent, and lexis). What appears to be a monolingual delay may be traced to monolingual learners gradually ‘distilling’ vocal pitch into its many communicative functions. The complexity of this process in tone languages may temporarily disfavor tone language learners. For non-tone language (e.g., English monolingual) learners, the division of labor carried by pitch is arguably more categorical: suprasegmental variation is more tightly bound to non-lexical functions and lexical contrast is marked by segmental variation. For Mandarin monolingual learners, the functions of suprasegmental variation are distributed over lexical and non-lexical functions, which may present a greater learning burden. So then why do bilingual learners of Mandarin and English not demonstrate effects of this burden? As discussed above, the presence of phonological conflict combined with a bilingual advantage for negotiating conflict may confer upon bilingual Mandarin–English learners early advantages less available to monolingual infants. This possibility is consistent with the bilingual advantage observed herein, but merits further empirical study. A second possibility derives from stimulus-specific effects. Each of the prior studies documenting tone integration in non-tone language learners (Graf Estes and Hay, 2015; Hay et al., 2015; Singh et al., 2015) used rising and falling tone contrasts (corresponding to Tones 2 and 4). These tones correspond closely to salient intonational categories in English and Mandarin, specifically, to the question/statement contrast (Singh and Chee, 2016). Young infants learning non-tone languages are astutely sensitive to the question/statement distinction (Geffen and Mintz, 2011; Frota et al., 2014), which serves an important pragmatic function in English as well as in Mandarin (Yuan, 2004, 2006). It is possible that these tone contrasts are integrated into lexical representations on account of their weighty pragmatic significance. One might expect tone contrasts that do not map directly onto intonational categories (such as those used in the present study) to be less salient to infants. It is possible that prior studies demonstrating tone integration in English learning infants engaged an extant sensitivity to intonational contrast, specifically, to the question/statement contrast. Sensitivity to this contrast in English learners may emerge earlier and may be more potent than sensitivity to native tones in Mandarin learners, although this account awaits empirical support. Third, it should be noted that Tone 3 is the most complex Mandarin tone on account of its bi-directionality (Gandour, 1983). It is acquired late relative to other Mandarin tones (Li and Thompson, 1977) and involves relatively complex laryngeal coordination (Wong, 2012). Tone 3 is also invoked in a common phonological alternation (Tone 3 Sandhi) resulting in context-driven substitutions to Tone 2. On account of these factors, the representation of Tone 3 in young learners may indeed be more fragile than that of other tones. Our design was predicated on infants having a well-specified representation of Tone 3 in order to detect deviations to Tone 1 and 2. Although speculative, further studies could examine stimulus-specific effects by using a different tone as the point of comparison and by exploring whether effects observed herein are symmetrical (i.e., whether a change from Tone 2 to Tone 3 would be more accurately detected at 12–13 months based on the possibility that Tone 2 sensitivity may profit from greater representational strength). A fourth possibility that is worth noting is that tone sensitivity may actually change between 12 and 14 months of age, a transition documented by Liu and Kager (2014). Liu and Kager (2014) observed that 11–12 months represented a comparative ‘low point’ in terms of infant tone sensitivity, which then progressively increased by 14–15 months. While their study was conducted with Dutch monolingual infants, it is conceivable that this trajectory may generalize to tone language learners. Although beyond the scope of the current paper, a replication of the current study at 14 months may allow for more direct comparisons between the present and previous studies.

Our primary purpose in conducting this study was to investigate bilingual infants’ negotiation of tone as a source of phonological conflict. Currently, there is mounting public interest in the science of bilingualism, perhaps inspired by the ever increasing numbers of children raised in bilingual environments (Peña and Bedore, 2010). However, parents and educators often wonder about the developmental effects of early bilingual exposure and specifically whether early exposure to two languages has the potential to confuse a young baby and consequently, to delay language development. These questions have garnered considerable popular and scientific attention. A recent suite of studies has demonstrated that infants may benefit from early exposure to two languages in a range of cognitive domains: learning sequences of information, imitation, anticipating events, visual habituation, and visual recognition memory (Kovács and Mehler, 2009a,b; Brito and Barr, 2014; Singh et al., 2014). However, an open question exists as to whether early bilingual exposure influences the uptake of each language. Previous research comparing monolinguals and bilinguals on the uptake of the formal properties of each language has focused predominantly on vocabulary size. These studies have suggested that single language vocabulary size is sometimes reduced in bilingual versus monolingual children (Bialystok and Feng, 2011; Hoff et al., 2012), although when measured across both languages, vocabulary size estimates can match or even surpass that of monolingual peers (e.g., Pearson et al., 1993; De Houwer et al., 2013). The current study adds to an ongoing narrative on whether two languages facilitate or confound the language-learning journey and suggests that an elemental formal property of bilingual development, acquisition of the native phonological systems, may benefit from bilingual exposure. Moreover, such advantages may be evident prior to the onset of a substantial productive vocabulary. Although prior studies have revealed bilingual advantages in learning the structure of languages, these studies have not typically assessed sensitivity to a property of language that causes cross-language conflict (e.g., Galambos and Goldin-Meadow, 1990; Campbell and Sais, 1995; Bialystok et al., 2014). Discursively, however, researchers have suggested that it may indeed be the presence of conflict that drives mastery of two systems, alluding to a direct relationship between incongruent language systems and gains in learning (Bialystok and Hakuta, 1994). This viewpoint is perhaps most famously exemplified by the now widely popularized statement by Bialystok and Hakuta that *“it is precisely because the structures and concepts of different languages never coincide that the experience of learning a second language is so spectacular in its effects.”* Providing one line of argument in support of this view, our findings invite the possibility that in some domains of bilingual development, cross-language conflict may not serve to confuse, but instead, to clarify.

## Conclusion

The title of this paper alludes to prior research positing ‘limits on bilingualism’ (Cutler et al., 1989; Dupoux et al., 2010). The postulate that there are limits on bilingualism is predicated on the notion that bilingual learners may never attain the degree of single-language proficiency exhibited by native monolingual speakers of the same two languages. In contrast to this hypothesis, the present study proposes that the early establishment of the phonological lexicon may be fortified by bilingual exposure. In contrast to bilingual infants, monolingually tone-exposed infants may follow a more protracted time course in determining the relationships between words and tones. Accordingly, mastery of two conflicting systems may potentially consolidate knowledge of the properties of each language, favoring phonological development in bilingual learners.

## Author Contributions

LS conceptualized the study, conducted data analyses, and drafted the manuscript. FP collected data for the study and drafted portions of the manuscript. CF collected data for the study, conducted data analyses, and drafted portions of the manuscript.

## Conflict of Interest Statement

The authors declare that the research was conducted in the absence of any commercial or financial relationships that could be construed as a potential conflict of interest.
