# Corrigendum: Limits on Monolingualism? A Comparison of Monolingual and Bilingual Infants' Abilities to Integrate Lexical Tone in Novel Word Learning

**DOI:** 10.3389/fpsyg.2016.00992

**Published:** 2016-06-30

**Authors:** Leher Singh, Felicia L. S. Poh, Charlene S. L. Fu

**Affiliations:** Department of Psychology, National University of SingaporeSingapore, Singapore

**Keywords:** lexical tone, phoneme discrimination, infant speech perception, Mandarin Chinese, word learning

Reason for Corrigendum:

Due to an oversight by the authors, Figures 3, 4 were switched. Figure 3 displays the results of Experiment 1 (Mandarin-English bilingual infants) and Figure 4 displays the results of Experiment 2 (Mandarin monolingual infants). The authors apologize for this mistake. This error does not change the scientific conclusions of the article in any way.

**Figure 3:**
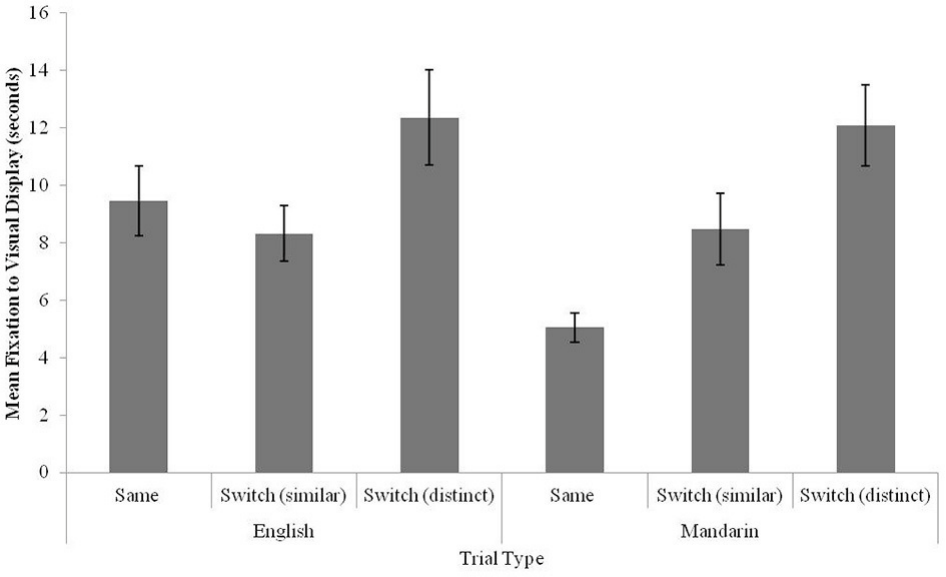
**Fixation times to the visual stimulus for Same, Switch (similar), and Switch (distinct) trials in 12-13-month-old bilingual infants (error bars: SEM)**.

**Figure 4:**
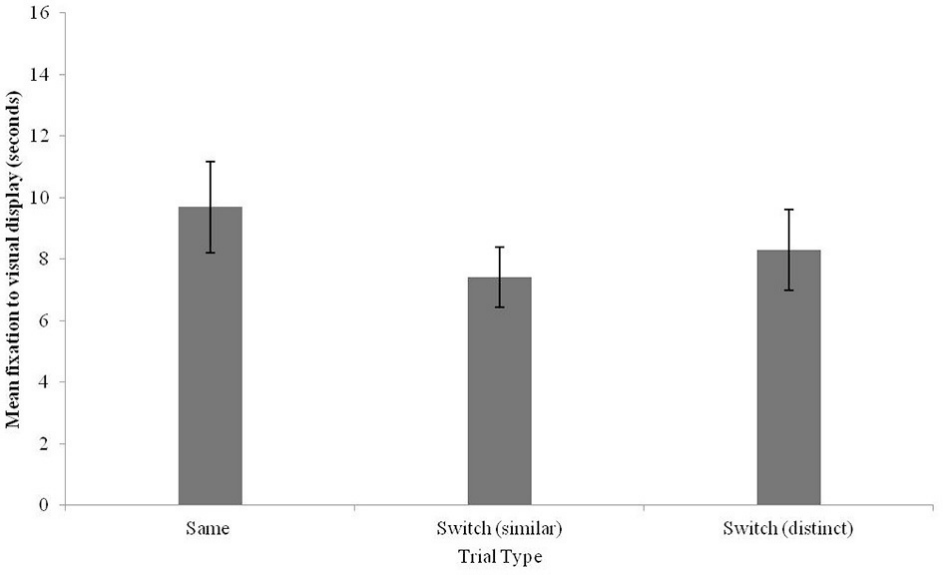
**Fixation times to the visual stimulus for Same, Switch (similar), and Switch (distinct) trials in 12-13-month-old Mandarin monolingual infants (error bars: SEM)**.

## Funding

This research was supported by a grant from the National University of Singapore, Singapore to LS (HSS R-581-000-178-646), to a Ministry of Education Tier 1 Academic Research Fund (FY2013-FRC2-009) grant to LS, and to a grant from the Singapore Children's Society to CF.

### Conflict of interest statement

The authors declare that the research was conducted in the absence of any commercial or financial relationships that could be construed as a potential conflict of interest.

